# Supplementary material for: Repeated Mild Head Injury Establishes a Senescent Cranial Bone Marrow Niche that Impairs Brain Metabolism
Source: bioRxiv. 2026 Jan 13:2026.01.12.699107. Preprint. [Version 1] doi: 10.64898/2026.01.12.699107 (PMC12871334; doi:10.64898/2026.01.12.699107)
Supplement: Supplement 1 [file NIHPP2026.01.12.699107v1-supplement-1.pdf]

bioRxiv preprint doi: https://doi.org/10.64898/2026.01.12.699107; this version posted January 13, 2026. The copyright holder for this preprint (which was not certified by peer review) is the author/funder, who has granted bioRxiv a license to display the preprint in perpetuity. It is made available under aCC-BY 4.0 International license.

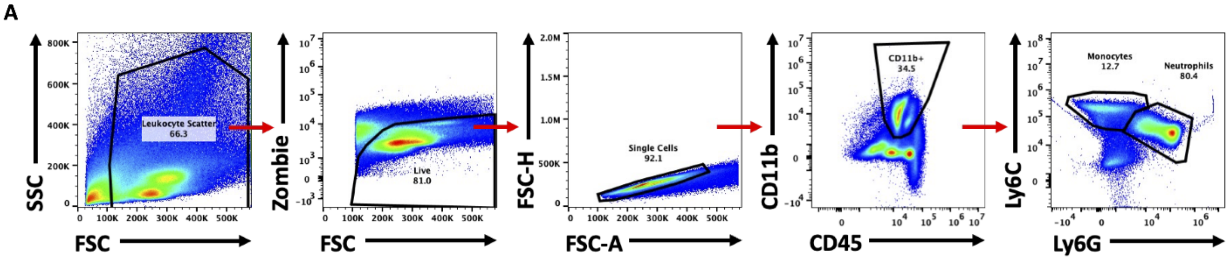

Supplemental Figure 1. Bone marrow cell gating strategy.

Viable singlet bone marrow leukocytes were identified using scatter properties and Zombie dye exclusion, followed by gating on $CD45^{+}CD11b^{+}$ myeloid cells. Monocytes were defined as $Ly6C^{+}$ $Ly6G^{-}$ cells, and neutrophils as $Ly6G^{+}$ cells. SSC side scatter, FSC-A forward scatter-area.
